# Editorial: Diversity and stability in aquatic plant communities

**DOI:** 10.3389/fpls.2025.1639349

**Published:** 2025-06-19

**Authors:** Igor Zelnik, Rui Pedro Rivaes, Mateja Germ

**Affiliations:** ^1^ Department of Biology, Biotechnical Faculty, University of Ljubljana, Ljubljana, Slovenia; ^2^ Marine and Environmental Sciences Centre, Faculty of Sciences, University of Lisbon, Lisbon, Portugal

**Keywords:** aquatic plants, macrophytes, wetland plants, rivers, lakes, wetlands, ponds, diversity

**Figure 1:**
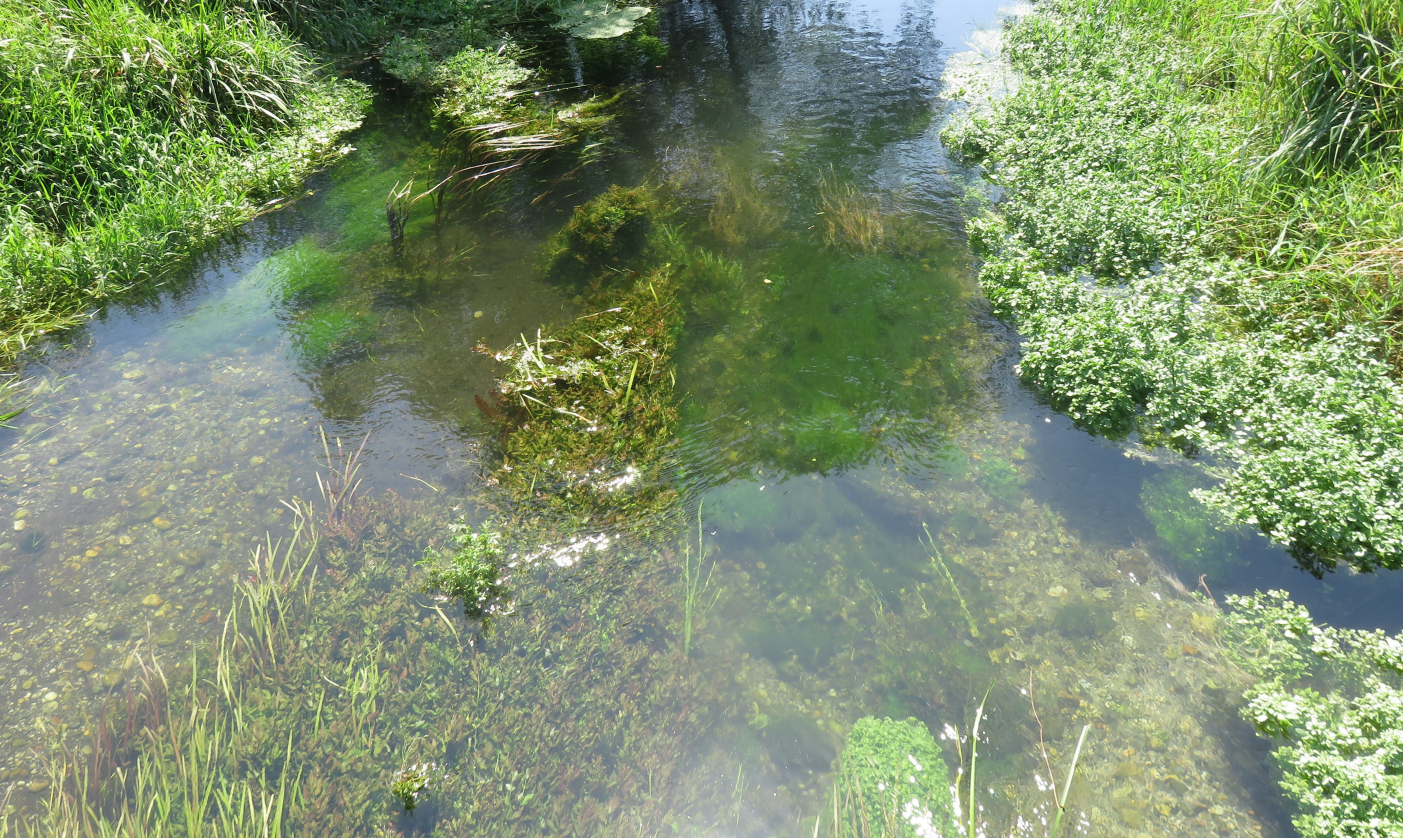
Diverse community of aquatic plants in the Struga river.

Freshwater ecosystems represent a small proportion of the terrestrial surface but host a very high proportion of biodiversity (Dudgeon et al., 2006). Nonetheless, these are facing a high rate of decline, both in biodiversity and in area (Zelnik and Germ, 2023). Biodiversity increases the temporal stability of communities and characteristics of the ecosystems through multiple mechanisms (Downing et al., 2014). Aquatic plants are fundamental for aquatic ecosystems (Zhou et al., 2023) and often govern the diversity and functionality of rivers, lakes, and wetlands (**Figure 1**). The mentioned issues can be found in the papers gathered in this Research Topic. Their findings deepen our understanding of freshwater ecosystem responses to environmental factors at different scales and gather important knowledge about management and climate change effects on the aquatic plant communities, their diversity and stability, protection and biological control of alien species.

Yang et al. showed temporal niche partition and traits tradeoff theory as primary strategies. The majority of the species pairs were niche partitioned and occurred either in spring or autumn. An approach using functional diversity provides quantitative information to explain macrophyte ecology more effectively than traditional methods based on taxonomy (Dalla Vecchia et al., 2020), since it is more resistant to environmental variations (Liu and Wang, 2018). The importance of functional diversity of aquatic plant communities in freshwater monitoring and conservation plans was highlighted by Stefanidis et al., quantifying this for Greek rivers while examining relationships with ecological factors. Growth forms and light preference were important traits that explained a large share of the total variance of functional composition. Functional richness was significantly higher at fine substrate systems and deep waters with low flow habitats.

Analogously, He et al. investigated the composition and diversity relationships between bacteria, fungi, and plant communities along a successional gradient behind receding glaciers. Taxonomic groups predicted the community composition more accurately than environmental factors, unlike taxa diversity, suggesting that the composition of one taxonomic group is not a strong driver of the diversity of another group. Additionally, Toth investigated the effect of epiphyton on foliar traits of a submerged rooted macrophyte *Potamogeton perfoliatus*, in a shallow lake, showing that epiphytic algal biomass influenced photophysiological traits of submerged macrophyte leaves. The complex interactions between epiphytes and submerged rooted macrophytes play an important role in habitat variability and overall ecosystem stability in littoral zones and should be considered in lake management. Klančnik et al. (2015) also showed the important role of the epiphytic diatoms in the prevention of adverse effects of short-wave radiation on submersed leaves of *Potamogeton perfoliatus*.

Furthermore, the spatiotemporal variability of the communities’ dynamics also influences coexistence, providing a biodiversity increase in variable conditions (Hallett et al., 2023). Xing et al. monitored the spatiotemporal changes in average wetland Normalised Difference Vegetation Index (NDVI) during the annual growing season in the Amur River basin for four decades, analysing wetland vegetation responses to climatic change. Under climate warming scenarios, the NDVI of a wetland will continue to increase, often resulting in lower diversity of plant communities. This is of great applicability as NDVI has been widely utilized for monitoring wetland vegetation vitality and distribution (Ojdanič et al., 2025).

Various factors shape the structure and diversity of aquatic plant communities, including several anthropogenic pressures, and should be considered in restoration planning. For instance, Svitok et al. explored the diversity of aquatic plants in rivers, streams, ponds and ditches in Central Europe and observed that ponds and ditches support higher macrophyte diversity than running waters. This highlights the conservation value of such artificial habitats and underscores the need to prioritize small waterbodies in conservation strategies. Another example is the effect of light conditions in the distribution of macrophytes (Liu et al., 2016), or water depth, determined by Yu et al. as the best predictor of nutrient threshold of submerged macrophytes collapse and recovery in lakes, further interacting with turbidity. Canopy-forming submerged macrophyte *Myriophyllum* sp*icatum* had a higher resistance to high nutrients and turbidity, whereas submerged macrophyte species richness had a significantly negative response to water depth. These results might provide quantitative guidance for lake restoration of diverse water depths.

Finally, environmental conditions changes influence the coexistence of plants, as Edwards et al. observed with the two common European wetland species *Carex acuta* and *Glyceria maxima*. Specifically, *C. acuta* was more affected by hydrologic changes, growing better in dry and saturated conditions, while *G. maxima* had a more positive response to fertilization. This confirms that it is crucial to maintain stable and diverse wetland plant communities to sustain ecosystem services like carbon sequestration and water purification. Furthermore, Toth recorded gradual changes within a season in the common reed *Phragmites australis* photosynthetic traits, comparing degraded and stable stands, suggesting a universal response to changing environmental conditions. Reed plants exposed to different levels of degradation showed comparable physiological plasticity, without a difference in trait variability between stands. This is likely to contribute to the resilience of reed plants by providing a wider range of adaptive traits under different conditions. The common reed is a very adaptable perennial grass growing in different wetlands, developing dense stands that enable soil stabilization and habitat provision (Ojdanič et al., 2025). Its dieback is a worldwide phenomenon occurring mainly due to water regulation and inadequate reed management practices.

Sediment accretion and nutrient addition also influence the growth of wetland plants and vegetative propagation, as Guo et al. observed, studying *Phalaris arundinacea* within a *Carex thunbergii* stand in the Yangtze River. An increased sedimentation rate facilitated the invasion of *P. arundinacea* into *Carex* stands, further enhanced by nutrient enrichment. Accordingly, management measures should consider sediment loads and nutrient inputs to prevent species invasion and maintain the ecological function of floodplain wetlands.

In conclusion, this Research Topic underpins the key role of aquatic plants in ecosystem stability and support of diversity in other biotic communities (O’Hare et al., 2018). Maintaining the diversity and stability of aquatic plant communities is paramount for the sustainability of freshwater ecosystems, and river restoration actions should not disregard this.
